# Pre-training Epidemic Time Series Forecasters with Compartmental Prototypes

**DOI:** 10.1145/3770855.3818928

**Published:** 2026-08-08

**Authors:** Zewen Liu, Juntong Ni, Bohan Wang, Max S. Y. Lau, Wei Jin

**Affiliations:** Emory University, Department of Computer Science, Atlanta, GA, USA; Emory University, Department of Computer Science, Atlanta, GA, USA; Emory University, Department of Computer Science, Atlanta, GA, USA; Emory University, Rollins School of Public Health, Atlanta, GA, USA; Emory University, Department of Computer Science, Atlanta, GA, USA

**Keywords:** Epidemic Forecasting, Time Series, Foundation Models, Compartmental Prototypes

## Abstract

Accurate epidemic forecasting is crucial for outbreak preparedness, but existing data-driven models are often brittle. Typically trained on a single pathogen, they struggle with data scarcity during new outbreaks and fail under distribution shifts caused by viral evolution or interventions. However, decades of surveillance data and the design of various compartmental models from diverse diseases offer an untapped source of transferable knowledge. To leverage the collective lessons from history, we propose CAPE, the first open-source pre-trained model for epidemic forecasting. Unlike existing time series foundation models that overlook epidemiological challenges, CAPE models epidemic dynamics as mixtures of latent compartmental population states, termed *compartmental prototypes*. It models a flexible dictionary of compartment prototypes directly from a large collection of simulation data, enabling each outbreak to be expressed as a time-varying mixture that links observed infections to latent population states. To promote robust generalization, CAPE adopts the next-token-prediction paradigm during pre-training with lightweight epidemic-aware regularization that aligns the learned prototypes with epidemiological semantics. On a comprehensive benchmark spanning 17 diseases, CAPE significantly outperforms strong baselines with zero-shot forecasting. This work represents a principled step toward pre-trained epidemic models that are both transferable and epidemiologically grounded. We provide our code in: https://github.com/nuuuh/CAPE.

## 1 Introduction

Infectious disease outbreaks pose a persistent threat to global public health and economic stability [29]. Effective outbreak management relies on accurate epidemic forecasting—the prediction of future cases, hospitalizations, and other critical metrics [1, 24, 42]. A wide range of models have been developed to provide these crucial forecasts, which generally fall into two categories. Knowledge-driven mechanistic models, such as the classic Susceptible-Infected-Recovered (SIR) [7] approach, are grounded in epidemiological principles; they divide the population into *compartments* that represent distinct *population states* (e.g., susceptible, infectious, recovered) and use differential equations to explicitly model flows among these states. In contrast, modern data-driven methods like LSTMs [37] learn complex patterns directly from historical data, offering greater flexibility without imposing a predefined structure of dynamics.

However, these data-driven forecasters are often trained for a single pathogen. This narrow scope makes them brittle: they face acute data scarcity during the critical early stages of a novel outbreak, and they fail under distribution shifts induced by viral evolution. While training across diverse pathogens and geographies proves to enhance downstream forecasting tasks [15], real-world data often lack of observation of hidden population groups (e.g., susceptible, exposed, vaccinated), which are essential for disclosing the underlying disease dynamics. Nevertheless, with decades of studies across various diseases, epidemiologists have uncovered diverse disease dynamics through mathematical modeling with extensive prior knowledge, which are often formulated as coupled ordinary differential equations, capable of simulating disease outbreaks. Motivated by the success of physics-informed neural networks in epidemic forecasting [35, 42], which make use of such prior knowledge, and large pre-trained models in language, vision, and time-series domains [20, 53], we ask: *Can we build a pre-trained epidemic forecaster that learns from the collective dynamics of infectious diseases to generalize effectively across real-world outbreaks?*

Simply applying a general time series foundation model [21] or training an epidemic-informed neural network is insufficient, as it overlooks core epidemiological challenges for generalization [20]: (1) *Structural heterogeneity*: Pathogens follow different effective compartmental progressions (e.g., SIR vs. SEIR [13]), so a single fixed mechanism cannot transfer broadly across diseases and regions. (2) *Hidden population states*: Surveillance data records only reported infections, while important states such as exposure, susceptibility, and immunity are not directly observed. These properties demand both large-scale and diverse simulations of diseases to capture various dynamics and a powerful epidemic pre-trained model structure that can adapt to different pathogens and provide forecasts under different hidden population groups.

### Our Solution.

*First*, we introduce **EpiRecipe**, a pipeline for building compartmental models and conducting large-scale simulations of diverse disease dynamics. *Second*, with the produced supervision data from EpiRecipe and real-world historical disease data, we introduce a novel pre-training-based epidemic model: CAPE (CompArtment Pre-training for Epidemics), which learns epidemic dynamics as a mixture of latent population groups, termed **compartmental prototypes**. To address structural heterogeneity and hidden states, rather than relying on a rigid, pre-defined structure, CAPE learns a flexible dictionary of prototypes directly from data via next-token prediction. This allows the model to transfer to downstream forecasting tasks by dynamically composing these prototypes via compartmental masking. Our contributions include:

1. **Large Scale Simulation Pipeline:** To enlarge the pre-training corpus, we propose **EpiRecipe**, a comprehensive simulation pipeline that maintains a catalog of 12 epidemiological compartments and over 20 transition dynamics to generate millions of structurally valid, diverse disease simulations
2. **Pre-training framework for epidemic time series forecasting:** We introduce the first open-source pre-training framework^1^ for epidemic forecasting. It learns latent compartmental prototypes directly from disease dynamics and provides uncertainty quantification by conducting stochastic structural inference via compartmental masking.
3. **Comprehensive evaluation benchmark and state-of-the-art performance:** We assemble a comprehensive evaluation pipeline spanning 17 diverse diseases in the US under an online forecasting setting. Even in a zero-shot setting, CAPE exhibits the best MSE and MAE against baselines and achieves a stronger performance compared to time series foundation models.
4. **In-depth analysis:** We conduct extensive analyses to uncover how pre-training improves representation learning. We provide the first evidence of *epidemic scaling laws* on simulation data and visualize how the model captures important epidemic properties like peak timing without explicit supervision.

## 2 Related Work and Problem Definition

### Epidemic Forecasting Models.

Traditionally, epidemic forecasting employs models like ARIMA [36], SEIR [13], and VAR [38]. ARIMA predicts infections by analyzing past data and errors, SEIR models population transitions using differential equations, and VAR captures linear inter-dependencies by modeling each variable based on past values. Recently, deep learning models [54], categorized into RNN-based, MLP-based, and transformer-based, have surpassed these methods. RNN-based models like LSTM [45], GRU [27], and more epidemic-specific models like EpiDeep [1] and EINNs [35] use gating mechanisms to manage information flow. MLP-based models use linear layers [50] or multi-layer perceptrons [4, 26, 28] for efficient data-to-prediction mapping and physics-informed distillation [43]. Transformer-based models [48, 55, 57] apply self-attention to encode time series and generate predictions via a decoder. However, these models are limited as they typically utilize data from only one type of disease without considering valuable insights from diverse disease datasets.

### Pre-trained Time Series Models.

To enable few-shot or zero-shot capabilities, transformer-based models often employ pre-training on large datasets, which typically use masked data reconstruction [33, 51] or promote alignment across different contexts [10, 49, 52]. For example, PatchTST [30] segments time series into patches, masks some, and reconstructs the masked segments. Larger foundational models like MOMENT [12] and Chronos [3] aim to excel in multiple tasks (e.g., forecasting, imputation, classification) and prove useful in epidemic forecasting [8, 14, 32], but training them requires substantial data and computational resources. A parallel line argues for epidemic-specific foundation models that pre-train on heterogeneous outbreak data while preserving epidemiological structure [20]. Kamarthi et al. [15] pre-train a model on various diseases, improving downstream performance and highlighting pretraining’s potential in epidemic forecasting. However, the complete implementation is not publicly available. Moreover, existing approaches overlook hidden compartmental influence and zero-shot ability in epidemic forecasting and lack a deep analysis of how pre-training materials impact downstream performance. In this study, we introduce latent compartment modeling and conduct a thorough analysis of these questions.

#### Problem Definition.

Given historical observations $x\in R^{T}$, the goal is to forecast future infections $y\in R^{H}$, where T and H denote the lookback window and forecast horizon, respectively. We employ a pre-training framework on a hybrid corpus ${𝒟}_{\text{pre}}={𝒟}_{\text{syn}}\cup {𝒟}_{\text{real}}$, comprising synthetic samples from EpiRecipe and historical real-world data. Adopting a *next-token-prediction* paradigm, we define a patching operator $𝒫:R^{T}\to R^{N\times P}$ that segments x into a sequence of N tokens $X^{\prime }=\left({x^{\prime }}_{1},\ldots ,{x^{\prime }}_{N}\right)$, where each ${x^{\prime }}_{i}\in R^{P}$ is a patch of length P. The model $f_{\theta }$ is trained to autoregressively predict the next token ${x^{\prime }}_{t+1}$ and latent epidemic states $z_{t+1}$ (e.g., compartmental dynamics, $R_{t}$) conditioned on the context ${x^{\prime }}_{1:t}$. The optimization objective is:

(1) $$\underset{\theta }{\text{min}}\;E_{x\sim {𝒟}_{\text{pre}}}\left[\sum_{t=1}^{N-1}\left({ℒ}_{\text{rec}}\left({\hat{x^{\prime }}}_{t+1},{x^{\prime }}_{t+1}\right)+\lambda {ℒ}_{\text{epi}}\left({\overset{ˆ}{z}}_{t+1},z_{t+1}\right)\right)\right]$$

where ${\hat{x^{\prime }}}_{t+1},{\overset{ˆ}{z}}_{t+1}=f_{\theta }\left({X^{\prime }}_{1:t}\right)$ are the predicted token and latent states, and λ balances the reconstruction loss ${ℒ}_{\text{rec}}$ with the epidemic consistency loss ${ℒ}_{\text{epi}}$.

## 3 Proposed Method

Our pre-training framework aims to overcome the core challenges of *structural heterogeneity* and *hidden population states* inherent in epidemic forecasting. We address these issues through two main contributions: (1) a comprehensive pipeline for producing random systems of disease dynamics (3.1), and (2) a flexible model architecture that learns latent compartmental prototypes directly from simulation data, with epidemic-aware pre-training objectives that guide the model to learn robust, generalizable representations (3.2). In addition, derived naturally from the model structure, we also introduce a stochastic structural inference for both effective adaptation to downstream tasks and uncertainty estimation (3.3).

### 3.1 EpiRecipe

**Figure 1:**
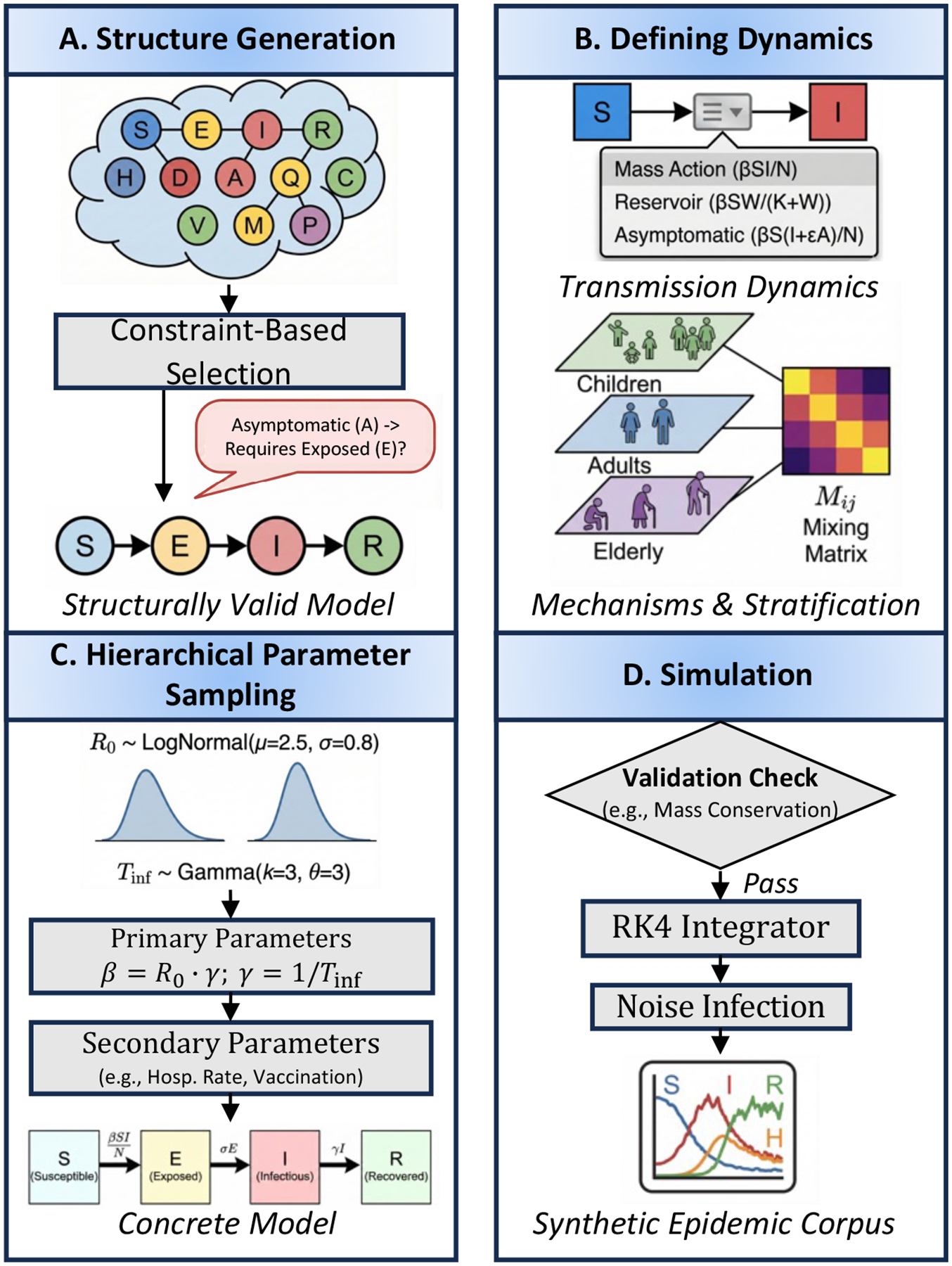
Pipeline of EpiRecipe.

A fundamental challenge in pre-training epidemic forecasting models is the scarcity and heterogeneity of real-world outbreak data. To address this, we develop **EpiRecipe**, a constraint-based simulation pipeline for generating diverse, epidemiologically valid compartmental models and their corresponding synthetic time series. This augments our pre-training corpus by exposing the model to a wide range of disease dynamics. The overall pipeline is shown in Figure 1 and more details are provided in Appendix A.2.

#### Constraint-Based Model Generation.

To ensure coverage of diverse compartment states and resolve the challenge of structural heterogeneity behind disease outbreaks, EpiRecipe maintains a catalog of 12 epidemiological compartments [5], representing diverse population groups (e.g., infectious individuals (I)):

(2) C={S,E,I,R,H,V,Q,D,P,W,A,C},

along with their dependency constraints encoded as requirements, defined as a mapping $f_{\text{req}}:𝒞\to 𝒫(𝒞)$, where each compartment maps to a subset of required prerequisite compartments 𝒫(𝒞). For instance, the Infectious (I) compartment requires the Susceptible (S) compartment to exist, i.e., $E\in f_{\text{req}}(A)$ [2].

#### Automatic Transition Construction.

Even with the same set of compartments, different diseases can have diverse transition dynamics. Therefore, once compartments are selected, EpiRecipe automatically constructs a transition network 𝒯 by randomly selecting from predefined mechanistic variants, where each transition from compartment i to j is governed by a flow function $f_{ij}$. For example, infection dynamics can follow standard mass-action $f_{S\to E}=\beta SI/N$ [18]. The pipeline selects appropriate variants of flow functions based on the available compartments, and the complete system evolves according to:

(3) $$\frac{dx_{c}}{dt}=\underset{\text{inflow}}{\underbrace{\sum_{i\to c}f_{ic}(x;\theta )}}-\underset{\text{outflow}}{\underbrace{\sum_{c\to j}f_{cj}(x;\theta )}},$$

where x is the state vector representing the population in each compartment, and the sums represent all incoming and outgoing transitions for compartment c.

#### Population Stratification.

Real epidemics exhibit heterogeneous dynamics across demographic groups. To capture this, EpiRecipe partitions the population into G groups (e.g., children, adults, elderly), following the implementation in CovidSim [46]. Each group g is a population fraction and has its own group-specific properties like transmission rate $\beta_{g}(t)$. Inter-group transmission is governed by a mixing matrix $M\in R^{G\times G}$ with predefined patterns (e.g., homogeneous or assortative). The stratified infection dynamics become: $f_{S\to I}^{\left(g\right)}=\beta_{g}\frac{S_{g}}{N_{g}}\sum_{h=1}^{G}M_{gh}\iota_{h}I_{h}$, where $S_{g},I_{g}$, and $N_{g}$ denote the susceptible, infectious, and total population in group g, respectively. This produces epidemic curves with realistic subpopulation dynamics and complex aggregate patterns. Specific sampling distributions for all multipliers are provided in Appendix A.2.

#### Hierarchical Parameter Sampling.

To ensure epidemiological realism, parameters are sampled hierarchically in three stages. *First*, primary epidemiological quantities, e.g., the basic reproduction number $R_{0}$, are sampled from informed prior distributions. *Second*, derived parameters are computed deterministically to maintain consistency, e.g., the baseline transmission rate $\beta_{0}=R_{0}\cdot \gamma$. Seasonal forcing is optionally applied via:

(4) $$\beta (t)=\beta_{0}\left[1+\varepsilon \;\text{cos}\left(\frac{2\pi t}{T_{\text{period}}}+\phi \right)\right],$$

where $\varepsilon ,T_{\text{period}}$, and ϕ control the amplitude, period, and phase of seasonal variation, respectively. *Lastly*, secondary parameters required by the selected transitions (e.g., hospitalization and vaccination rates) are sampled from predefined ranges. All sampling distributions and ranges are provided in Appendix A.2.

#### Validation and Simulation.

Each generated model undergoes validation to ensure: (1) population conservation across living compartments, (2) no dead-end states, i.e., every non-terminal compartment has at least one outflow transition, and (3) existence of a valid infectious pathway from S to I in the transition graph. Valid models are then integrated using a fourth-order Runge-Kutta (RK4) solver, and Gaussian observation noise is injected to mimic real-world reporting imperfections:

(5) $$\{x(t){\}}_{t=0}^{T}=\text{RK}4\left(\frac{dx}{dt},x_{0},T\right),\;\tilde{x}(t)=\text{max}(x(t)+\eta (t),0),$$

where $\eta (t)\sim 𝒩\left(0,\lambda^{2}\cdot \text{diag}(\text{Var}(x))\right)$ and λ controls the noise level. Through this process, EpiRecipe generates millions of samples with varied compartmental structures, transition mechanisms, and parameter regimes, providing the diverse pre-training corpus essential for learning generalizable patterns.

### 3.2 Model Structure for Epidemic Pre-training

Similar to the current large language models and foundation models in time series [3], CAPE conducts next-token-prediction in an autoregressive way. CAPE is composed of three major components: (1) Autoregressive temporal encoder $f_{\text{enc}}$, (2) Compartmental decoding $f_{\text{comp}}$, and (3) Masked multi-groups attention $f_{\text{dec}}$.

#### Autoregressive Temporal Encoder $\left(f_{\text{enc}}\right)$.

To effectively capture the evolving momentum and latent inertia of an outbreak, it is important to distill raw, often non-stationary infection trends into a high-dimensional temporal context that preserves both local fluctuations and global epidemiological trajectories. Given an observed infection trajectory $x\in R^{T\times 1}$, *smoothing* [6] and *patching* [30] is performed to formulate the denoised token sequence $x^{\prime }\in R^{N\times t}$, where N is the number of tokens and t=T/N is the token size. Then, an autoregressive model (e.g., LSTM, TCN, or Transformer) is applied to encode temporal information, producing embeddings for each patch $h_{i+1}=f_{\text{enc}}\left(x_{0}^{\prime },\ldots ,x_{i}^{\prime }\right)\in R^{D}$, where i denotes the token index and D is the embedding size. This encoding formulates the temporal context of past infections $H\in R^{N\times D}$.

#### Compartmental Decoding $\left(f_{\text{comp}}\right)$.

Decoding directly from the univariate context is insufficient, as it not only ignores the provided supervision signals from different compartments but also the influence of hidden compartments on the forecast of future infections. Therefore, we propose to decode compartmental context in the latent space with both temporal context H and a codebook [56] of learnable compartmental prototypes, denoted as $Z\in R^{|C|\times D}$, where C is a set of compartments that match with the ones in EpiRecipe. Finally, we use cross-attention to produce the contextualized compartment embeddings as:

(6) $$H^{\prime }=f_{\text{comp}}\left(H,Z\right)=\text{softmax}\left({ZH}^{⊤}/\sqrt{D}\right)\;H\in R^{|C|\times N\times D}.$$

Eventually, the prediction of the next token is decoded from the corresponding compartment embedding.

#### Masked Multi-groups Attention $\left(f_{\text{dec}}\right)$.

The observed dynamics of a compartment are often the result of complex, unobserved interactions among multiple latent population groups (e.g., age, occupation, or mobility-based strata) with differing contact rates and vulnerabilities. To bridge the gap between these hidden population states and visible compartmental outcomes, we propose *Masked Multi-groups Attention*. By implicitly modeling these heterogeneous group dynamics through the regularization of the effective reproduction number $R_{t}$ [22], where $R_{t}>1$ signals expansion and $R_{t}<1$ signals decline of infections, we enforce an epidemiologically coherent latent space that ensures the model’s predictions align with the physical laws of disease transmission. Overall, this process produces predictions $\text{Y}=f_{\text{dec}}\left(H^{\prime },M\right)$ with a compartment mask M, as detailed below.


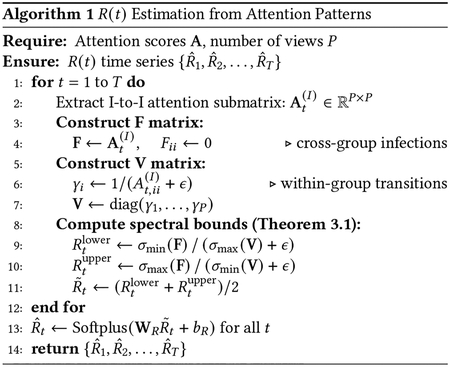


*First*, each compartment $H_{C_{i}}^{\prime }$ is projected to P multiple groups via linear projections ${\overset{ˆ}{H}}_{C_{i},j}=W_{j}H_{C_{i}}^{\prime }$, each corresponding to a population group. Such an idea is similar to the linear projections in computing query, key, and value in self-attention and the mappings in multi-head attention [41], which enhances the expressiveness of the model. *Second*, giving the mask **M**, the masked self-attention is applied to model the interactions among compartments of interest in the latent space, producing $\bar{H}=\text{MaskedAttention}(\overset{ˆ}{H},M)$. *Third*, with the attention scores indicating the strength of contributions/interactions, we regularize the attention scores within the infectious group to match the actual dynamics. To bridge the gap between these latent attention patterns and $R_{t}$, we map the learned attention matrix to the components of the Next-Generation Matrix [9], where off-diagonal entries correspond to cross-group transmission rates (matrix F) and diagonal entries reflect within-group stability (matrix V). Consequently, Theorem 3.1 provides the spectral bound for estimating $R_{t}$ (Proof in Appendix A.4).

**Theorem 3.1** (Bounds for $R_{t}$). Let $F,V\in R^{n\times n}$ *with* V *invertible, and define* $R_{t}\equiv \rho \left({FV}^{-1}\right)$, *where* ρ(⋅) *is the spectral radius. Then*:

(7) $$\frac{\sigma_{\text{min}}(F)}{\sigma_{\text{max}}(V)}\leq R_{t}\leq \frac{\sigma_{\text{max}}\left(F\right)}{\sigma_{\text{min}}\left(V\right)},$$

*where* $\sigma_{\text{max}}(\cdot )$ *and* $\sigma_{\text{min}}(\cdot )$ *denote the maximal and minimal singular values, respectively*.

Based on Theorem 3.1, we derive a differentiable estimation of ${\overset{ˆ}{R}}_{t}$ from the attention scores via Algorithm 1, and match the estimation with ground truth using ${ℒ}_{R_{t}}=\text{MSE}\left({\overset{ˆ}{R}}_{t},R_{t}\right)$. Lastly, for each compartment $C_{i}$, the latent groups are aggregated and projected to the next token via $Y_{i}=\text{MLP}\left(\sum_{j=1}^{P}{\overset{‾}{H}}_{C_{i},j}\right)$, where $Y_{i}$ is the future trajectory of compartment $C_{i}$.

### 3.3 Epidemic Pre-training and Inference

#### Pre-training.

We propose a dual-stage pre-training strategy, where the first stage is to inject epidemic inductive bias into the model via large-scale simulation data, and the second stage is to adapt to real-world data. During the first stage of pre-training, for each sample, EpiRecipe provides ground truth for (1) the next token of all compartments and the $R_{t}$, and (2) a binary compartment mask M to block the irrelevant compartments. Therefore, the loss function is formulated as:

(8) $$ℒ=\sum_{i}^{\left|C\right|}\alpha_{i}M_{i}\cdot \text{MSE}\left(Y_{i},{\overset{ˆ}{Y}}_{i}\right)+\beta {ℒ}_{R_{t}},$$

where α and β are weights to control the alignment with the ground truth compartments and the underlying effective reproduction number. In the second stage, we collect historical infections from different diseases and conduct the same pretraining strategy while only activating S and I compartments and without applying ${ℒ}_{R_{t}}$.

#### Stochastic Structural Inference.

While pre-training provides labels for each compartment and the compartment mask, *structural heterogeneity* and *hidden population states* remain challenges for downstream real-world samples. Therefore, during inference on downstream datasets, CAPE provides predictions with uncertainty by predicting K times while randomly masking each compartment with a probability of 50% (Basic compartments like S and I will never be masked). This process implicitly samples K distinct epidemic model structures, producing a distribution of plausible forecasts. The final forecast is derived by aggregating these realizations (e.g., pooling for point prediction and quantiles for uncertainty quantification), which effectively mimics an ensemble of diverse compartmental models [34].

## 4 Experiment

### 4.1 Setup

#### Datasets.

*Pre-train Corpus:* Our pipeline streams samples on-the-fly (avoiding large pre-compiled datasets), padding to match varying series lengths. We treat 4 time steps as one token (one month) and pre-train on approximately 240M tokens, followed by in-domain finetuning on 7 distinct real-world diseases at the same token size. *Downstream Evaluation:* We collect 17 diverse diseases from Project Tycho [40] with outbreaks across US states (per-dataset statistics in Table 3), using an online setting [35] (first 30% for base training, remainder for evaluation). More details are in Appendix A.5.

#### Baselines.

Using the *EpiLearn* toolkit [23], we compare against two categories. *Non-pretrained*: RNN-based epidemic forecasters EpiDeep and EINNs [1, 35], the MLP-based DLinear [50], and transformer-based PatchTST [15, 30]. *Pre-trained*: PEM [15] and the time series foundation models MOMENT [12], CHRONOS [3], and Moirai [47]. We also compare against per-sample statistical models ARIMA [31] and SIR [7]. All baselines are tuned per fold by grid search over hidden sizes {128, 512}, layers {2, 3}, and learning rates {0.001, 0.005}, while CAPE is zero-shot and tunes only the smoothing window and ensemble strategy on validation data (Appendix A.6).

#### Experiment Setting.

We adopt an online (rolling) protocol that respects temporal order and avoids future leakage: the first 30% of each tokenized series is the initial training window, and the remaining 70% forms non-overlapping folds, each using an expanding window to predict one output token (4-week horizon) from eight input tokens (32 weeks). Because each model class suits a different data regime, we benchmark across three paradigms. (i) **Full-shot**: data-driven baselines (e.g., GRU, EINN, EpiDeep) are retrained on all data up to each fold boundary, since they are brittle and degrade without recent observations [44]; (ii) **Zero-shot**: general foundation models (Chronos2, Moirai, MOMENT) forecast without fine-tuning, which can otherwise degrade their generalization under distribution shift [19, 25]; and (iii) **Few-shot**: a new paradigm for ensembling-based forecasters (e.g., CAPE) that uses the current fold’s validation split to select the ensemble aggregation strategy. CAPE bridges these regimes by pre-training on the *EpiRecipe* corpus and replacing rigid fine-tuning with stochastic structural inference via compartmental masking, yielding calibrated uncertainty and robust point forecasts across pathogens.

#### Research Questions.

In the following experiments, we propose and answer the following questions: ***Q1:*** How does CAPE compare against state-of-the-art non-pretrained and pretrained baselines? ***Q2:*** What is the contribution of each component in CAPE’s architecture? ***Q3:*** What is CAPE learning from the vast pre-training corpus? ***Q4:*** Do epidemic forecasting models exhibit scaling laws similar to those observed in language models? ***Q5:*** What’s the impact of pre-training on forecasting downstream real-world diseases? Specifically: (1) How does the diversity of pre-training data affect downstream performance? (2) What is the relationship between pre-train compute and downstream improvement? (3) Can CAPE learn meaningful disease representations? (4) Can CAPE produce uncertainty quantification?

### 4.2 Comparison with Baselines

To answer **Q1**, we benchmark CAPE against both full-shot and zero-shot baselines across 17 diseases, demonstrating state-of-the-art performance in both settings.

#### Comparison with Full-shot Models.

As shown in Table 1, both CAPE variants, pre-trained solely on synthetic data (CAPE_*zero*_) and with additional in-domain finetuning on real-world surveillance data (CAPE), outperform the best baseline on 11/17 diseases in MSE and 10/17 in MAE, without finetuning on the training set. CAPE achieves the best overall performance (MSE: 0.154, MAE: 0.184), substantially outperforming the strongest baseline ARIMA (MSE: 0.235, MAE: 0.198). All deep learning baselines struggle in this data-scarce regime, with several exhibiting catastrophic failures. CAPE’s advantages are most pronounced on vaccine-preventable diseases (e.g., Measles, Mumps, Pertussis, Poliomyelitis), reducing MSE by 20–30% over the best baseline, suggesting that compartmental pre-training effectively captures nonlinear transmission dynamics. ARIMA retains an edge on trend-dominated diseases (e.g., Typhoid Fever), while Influenza remains challenging due to sharp seasonal spikes.

#### Comparison with Zero-shot Foundation Models.

**Figure 3:**
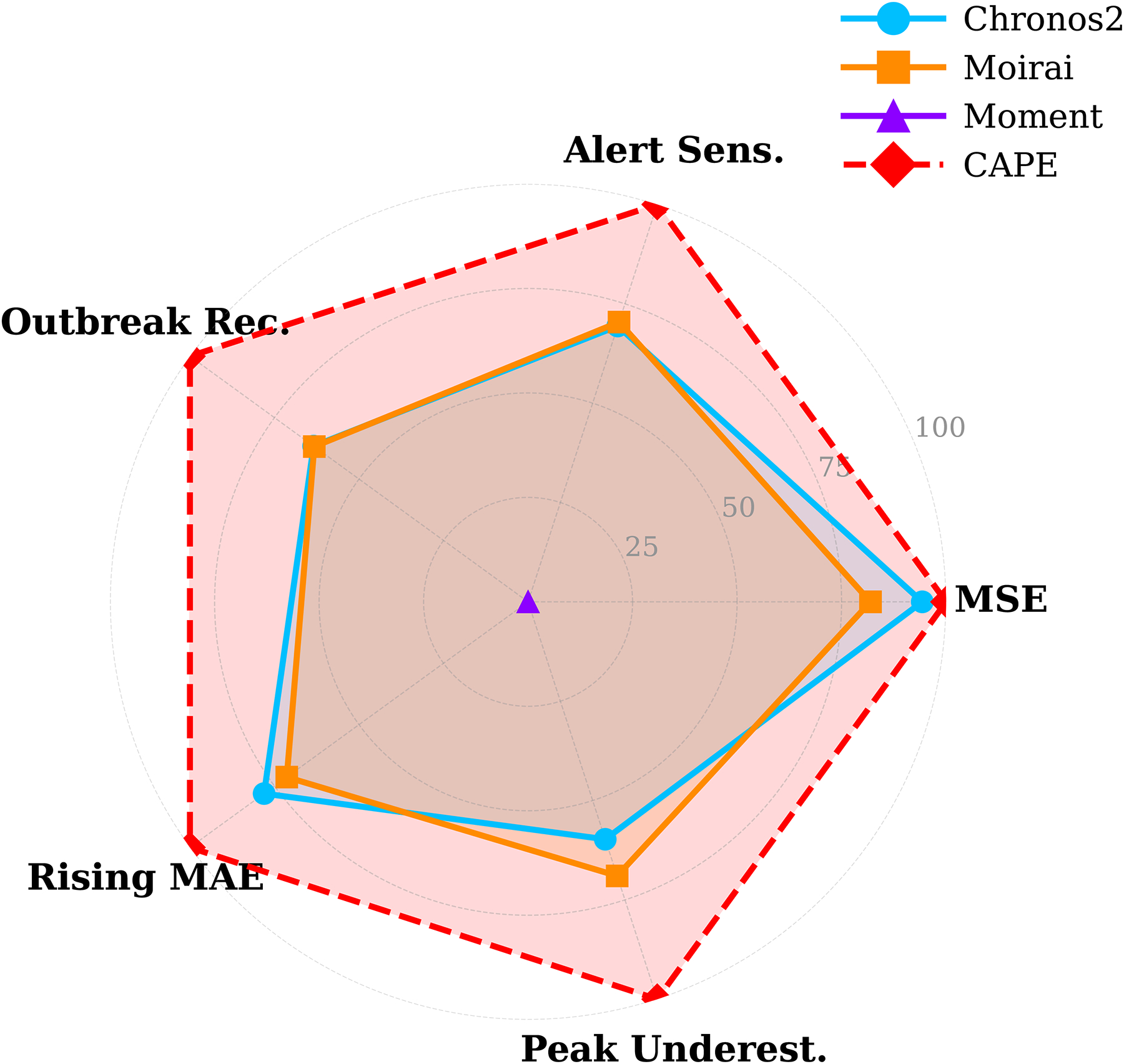
Comparison with foundation models across all datasets, on MSE and four epidemiologically critical metrics (defined in §4.2).

We further compare CAPE against state-of-the-art time series foundation models, e.g., Chronos2 [3], Moirai [47], and Moment [12], in the zero-shot setting. Beyond MSE, we report three epidemiologically critical metrics (with ${\overset{ˆ}{y}}_{i}$ the prediction and $y_{i}$ the truth): *Outbreak Sensitivity*, the recall of high-value detection $\left|\left\{i:{\overset{ˆ}{y}}_{i}>\tau ,y_{i}>\tau \right\}\right|/\left|\left\{i:y_{i}>\tau \right\}\right|$ at a moderate $\left(\tau =\text{median}(y)+0.5\sigma_{y}\right)$ and a strict $\left(\tau =\mu_{y}+\sigma_{y}\right.$, “Alert”) threshold; *Rising Phase MAE*, the MAE over growth-phase samples $\left(y_{i}-x_{i}^{\text{last}}>0.1\right)$; and *Peak Underestimate Rate*, the fraction of peak values $\left(y_{i}\geq P_{90}(y)\right)$ underestimated. As shown in Figure 3, CAPE achieves the lowest MSE and Rising Phase MAE, demonstrating superior point forecast accuracy. More importantly, CAPE excels on the epidemiologically critical metrics with the highest Alert Sensitivity (78.35% vs. 64.21% for Moirai), Outbreak Recall (86.65% vs. 74.04% for Moirai), and the lowest Peak Underestimate Rate (55.29% vs. 66.99% for Moirai), indicating better detection of emerging outbreaks and the power of pre-training with domain-specific synthetic data.

### 4.3 Ablation Study

To answer **Q2**, we evaluate the contribution of each proposed component through an ablation study over 6 diseases, using the entire time series as the test set. We progressively add components on top of a no-pre-training baseline: (i) pre-training (on real-world or naive single-compartment synthetic data), (ii) compartmental decoding, and (iii) masked multi-groups attention. As shown in Table 2, pre-training with naive synthetic data (only the infectious compartment) achieves comparable performance to pre-training with real-world epidemic data (47.3% vs. 46.2% average improvement), demonstrating that synthetic pre-training can effectively substitute for scarce real-world outbreak data. Adding compartmental decoding provides the most substantial marginal improvement (61.8%), validating our hypothesis that explicitly modeling latent compartmental states helps capture hidden transmission dynamics, with pronounced gains on diseases with complex dynamics such as Gonorrhea (92.3%) and Rubella (76.8%). The masked multi-groups attention contributes a further 7.3% average improvement, with notable gains on Tuberculosis (25.3%) and Gonorrhea (22.4%), diseases known to exhibit heterogeneous transmission across demographic groups [17, 39]. Together, these components reduce the average MSE from 1.987 to 0.371, an overall improvement of 81.3%, with each component addressing a distinct epidemiological challenge.

### 4.4 Learning from Simulation Data

**Figure 4:**
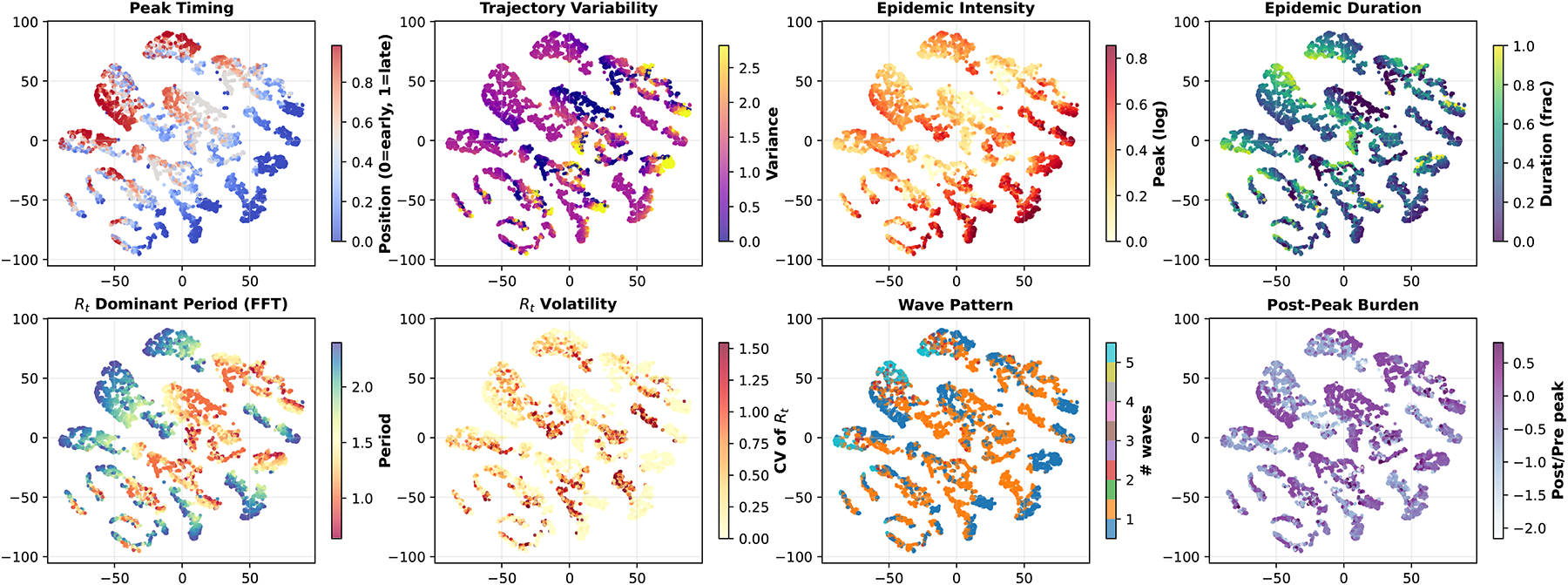
t-SNE visualization of CAPE encodings of synthetic EpiRecipe trajectories, colored by eight epidemiological properties: peak timing $\left(t^{*}/T\right)$, trajectory variability, epidemic intensity $\left({\text{log}}_{10}\left(I^{*}+1\right)\right)$, epidemic duration (fraction of time above $0.1I^{*}),R_{t}$ dominant period (from the FFT of $R_{t}),R_{t}$ volatility (CV), wave-pattern count, and post-peak burden, where I(t) is the infection series with peak time $t^{*}$ and peak value $I^{*}$. CAPE organizes trajectories into coherent local clusters that correlate strongly with these properties: smooth gradients for continuous properties (e.g., peak timing, $R_{t}$ dominant period) and distinct groupings for discrete ones (e.g., wave patterns). This structure emerges purely from next-token prediction on diverse compartmental simulations, without explicit supervision on any property.

To answer **Q3**, we investigate the representations learned by CAPE and find that EpiRecipe not only produces infection trajectories with diverse epidemic properties, but also enables CAPE to learn good representations of these properties from the synthetic data. Specifically, we extract patch embeddings from 10,000 synthetic epidemic trajectories generated by EpiRecipe and visualize them using t-SNE, colored by eight epidemiological properties (defined in the caption of Figure 4), as shown in Figure 4. The visualization reveals that CAPE learns to organize epidemic trajectories into coherent local clusters that correlate strongly with meaningful epidemiological properties. Notably, the embeddings exhibit smooth gradients for continuous properties (e.g., peak timing, $R_{t}$ dominant period) and distinct groupings for discrete properties (e.g., wave patterns), suggesting that CAPE captures both fine-grained temporal dynamics and higher-level structural patterns. Crucially, this organization emerges purely from next-token prediction on diverse compartmental simulations, *without any explicit supervision* on these properties, demonstrating that the pre-training objective naturally induces epidemiologically meaningful representations that transfer to downstream forecasting.

### 4.5 Epidemic Scaling Law

**Figure 5:**
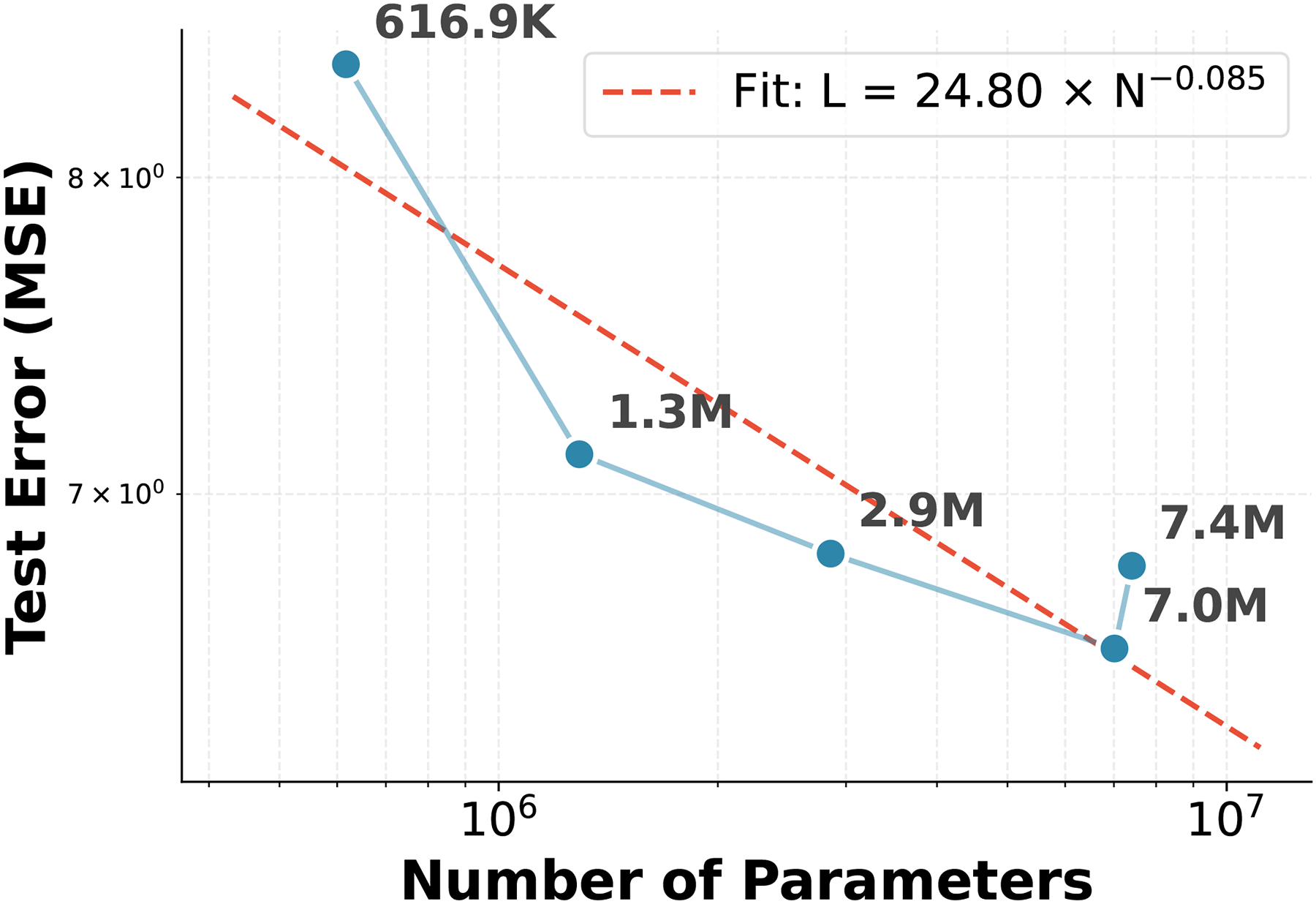

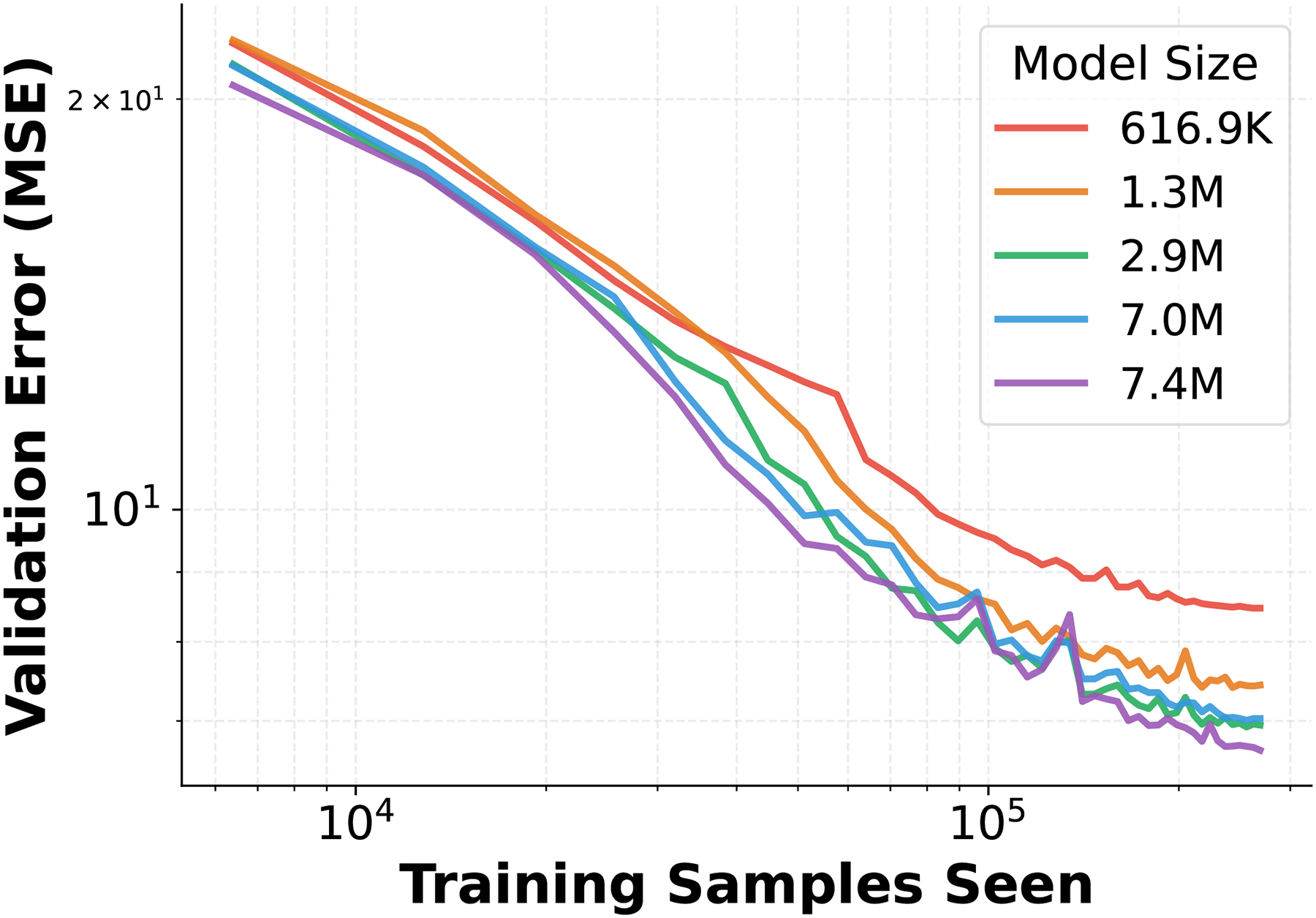
Scaling under model size and pre-train compute.

To answer **Q4**, we investigate whether neural scaling laws [16], well-established in language and vision domains, also hold for epidemic forecasting. To our knowledge, this is the first study of scaling behavior in epidemic pre-training, and we find that CAPE models pretrained on EpiRecipe-generated simulations exhibit consistent scaling along both model size and training-sample axes. **(a) Scaling with Model Size.** As shown in Figure 5a, test error decreases following a power law $L=24.80\times N^{-0.085}$ as the number of parameters N increases. The exponent −0.085 indicates slow but consistent improvement, falling within the range observed for natural language (−0.05 to −0.10) [16]. The shallow slope suggests epidemic dynamics have lower intrinsic dimensionality than language, so gains come more from architectural inductive bias (e.g., compartmental decoding) than from raw parameter count. **(b) Scaling with Pretraining Compute.** Figure 5b reveals that all model sizes exhibit consistent error reduction as training samples increase, with larger models achieving lower asymptotic error. Interestingly, smaller models (617K–1.3M) show faster initial convergence but plateau earlier, while larger models (7.0M–7.4M) continue improving with more data. This indicates that model capacity and data scale must be balanced: over-parameterized models require sufficient training samples to realize their potential, whereas under-parameterized ones saturate regardless of additional data. Practically, these trends let practitioners extrapolate the compute needed to reach a target accuracy before committing to expensive large-scale pre-training.

### 4.6 From Simulation to Reality: Impact of Pre-training on Downstream Forecasts

To answer **Q5**, we explore the effect of epidemic pretraining from both diversity and compute perspectives, and quantified the impact of pre-training over downstream performance, representation quality, and enabling uncertainty estimation.

#### (a) Impact of Data Diversity on Cross-diseases Transferability.

**Figure 6:**
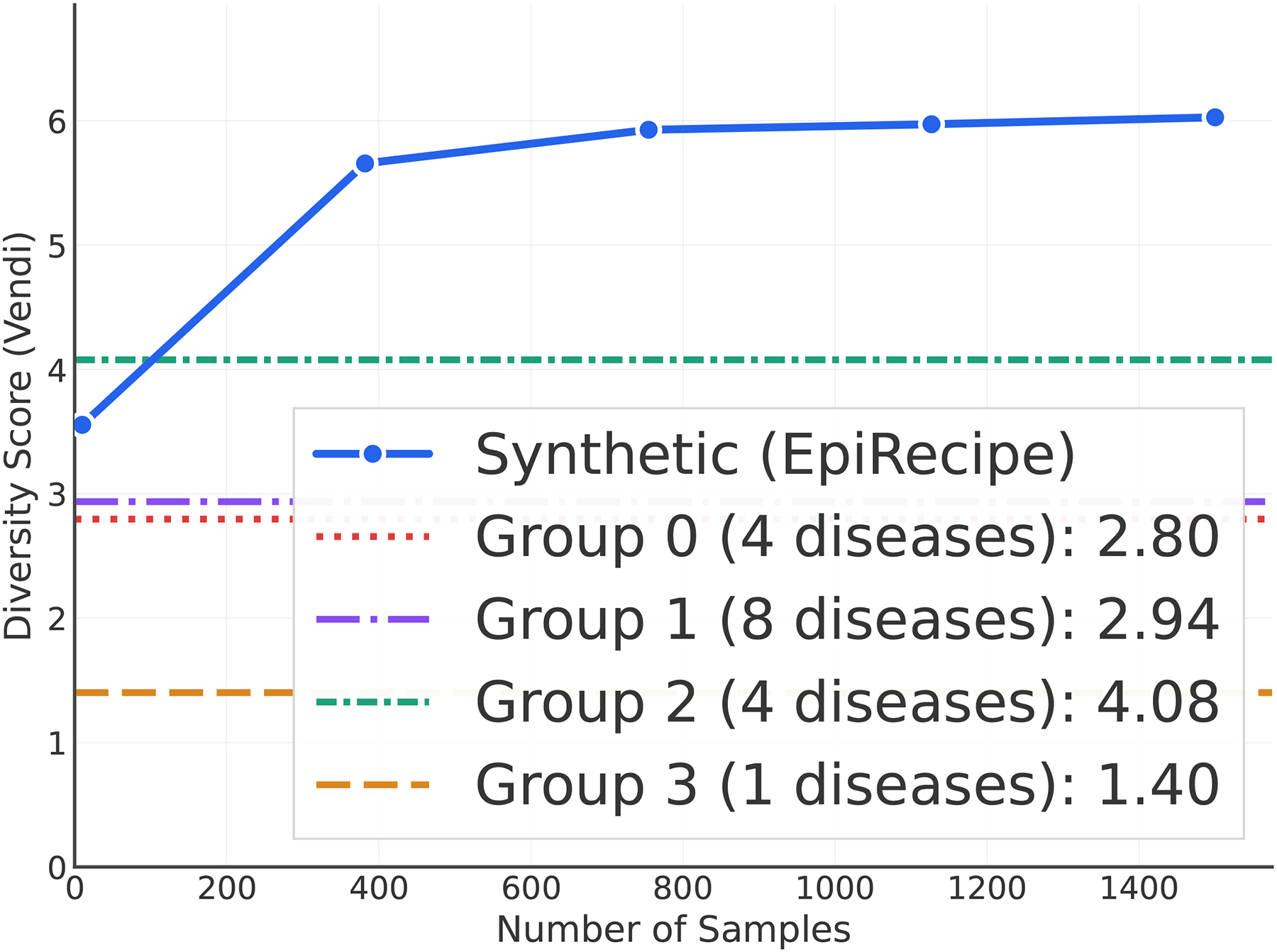

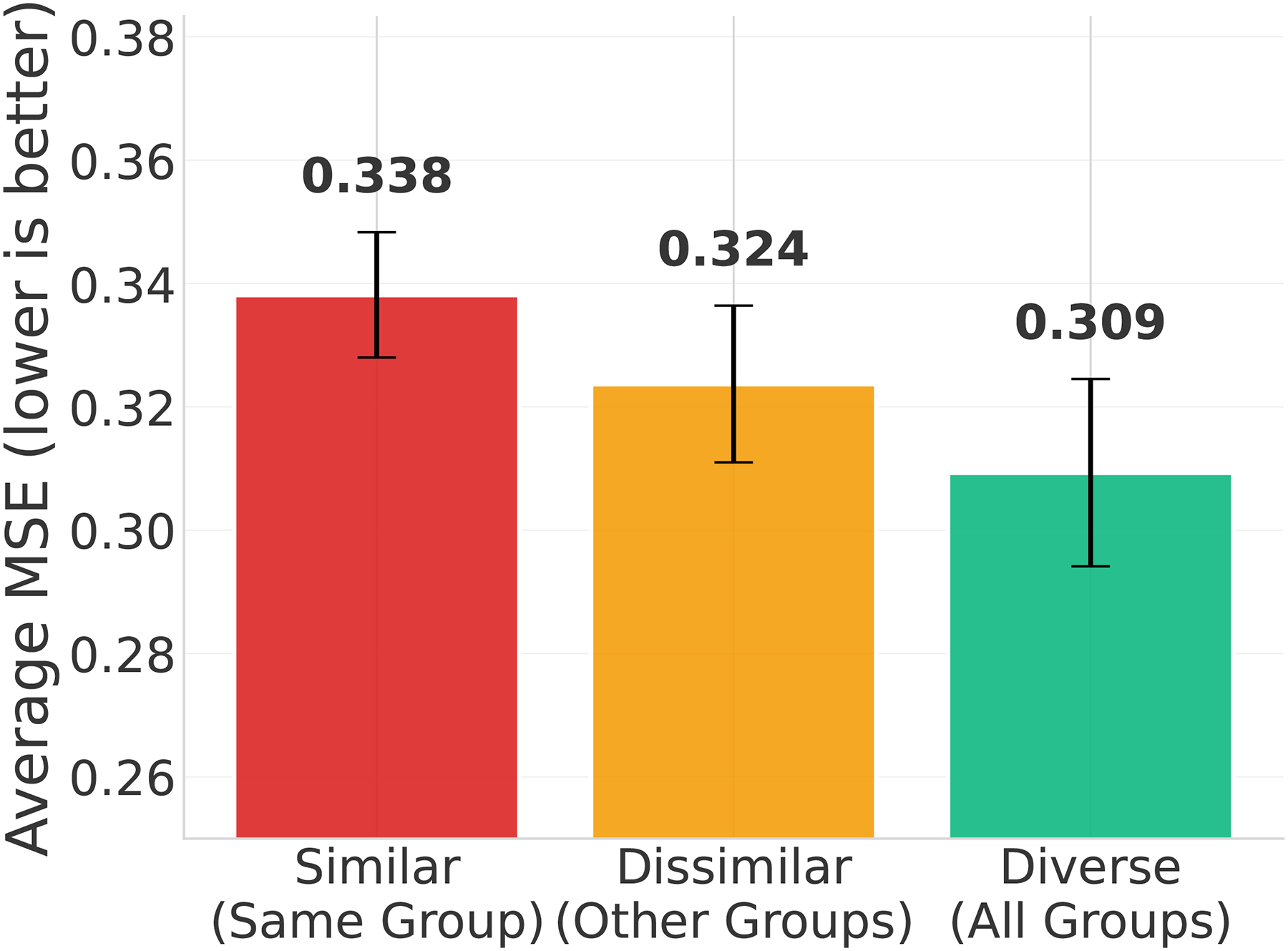
Diversity scores and transferability experiment.

We hypothesize that pre-training data diversity, rather than similarity to the target domain, is the key driver of downstream performance. To test this, we cluster 17 diseases from the Tycho dataset into 4 groups based on trajectory features (peak timing, asymmetry, autocorrelation, etc.) using hierarchical clustering, and measure diversity using the Vendi Score [11], which computes the diversity of elements in a dataset based on eigenvalue entropy of the similarity matrix. To isolate the effect of diversity from data quantity, we conduct a controlled experiment: for each target group, we pre-train on (1) the *same* group (similar), (2) *other* groups excluding the target (dissimilar) group, or (3) *all* groups (most diverse), with the sample count held constant across conditions so that any difference is attributable to diversity rather than scale. As shown in Figure 6b, pre-training on diverse data (All Groups: 0.309 MSE) consistently outperforms pre-training on similar data (Same Group: 0.338 MSE), an 8.6% relative improvement. Strikingly, even pretraining on *dissimilar* data (Other Groups: 0.324 MSE) outperforms the similar condition, indicating that exposure to varied dynamics is more valuable than proximity to the target distribution. This implies that a model pre-trained on a broad spectrum of *unrelated* diseases can still transfer effectively to a novel pathogen, having learned general mechanisms of epidemic growth and decay rather than disease-specific shapes; it also explains why CAPE’s synthetic corpus is so effective, as Figure 6a confirms EpiRecipe’s Vendi Score exceeds that of any single real-world disease group.

#### (b) Pre-training Compute vs Downstream Performance.

**Figure 7:**
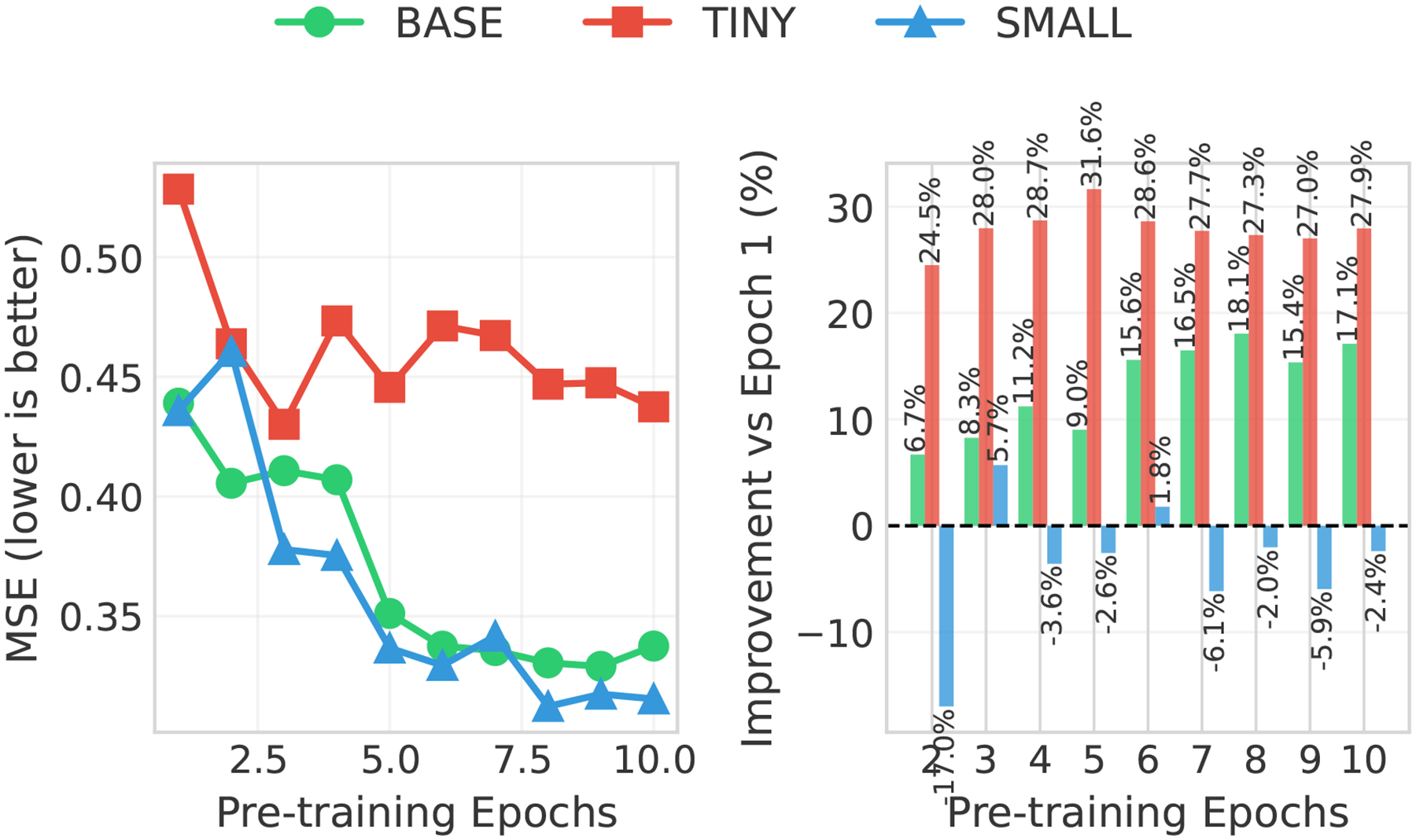
Pre-training compute vs downstream performance. Left: Zero-shot MSE on the Influenza data. Right: Relative improvement over epoch 1 across all diseases.

We investigate how pre-training computation affects downstream zero-shot performance by evaluating CAPE checkpoints saved at different epochs on 8 diseases from the Tycho dataset. Figure 7 (left) shows that MSE decreases monotonically with additional pre-training epochs across all model sizes on the Influenza data. Notably, most improvement occurs in the first 5 epochs, after which gains diminish, consistent with the scaling law findings. Figure 7 (right) quantifies the average relative improvement over epoch 1 across all diseases. The BASE model achieves consistent improvements of 15–28% throughout training, while smaller models like TINY and SMALL do not show consistent gains or decrease after two epochs, indicating larger models may benefit more from additional compute without overfitting.

#### (c) Learning of Disease Representations.

**Figure 8:**
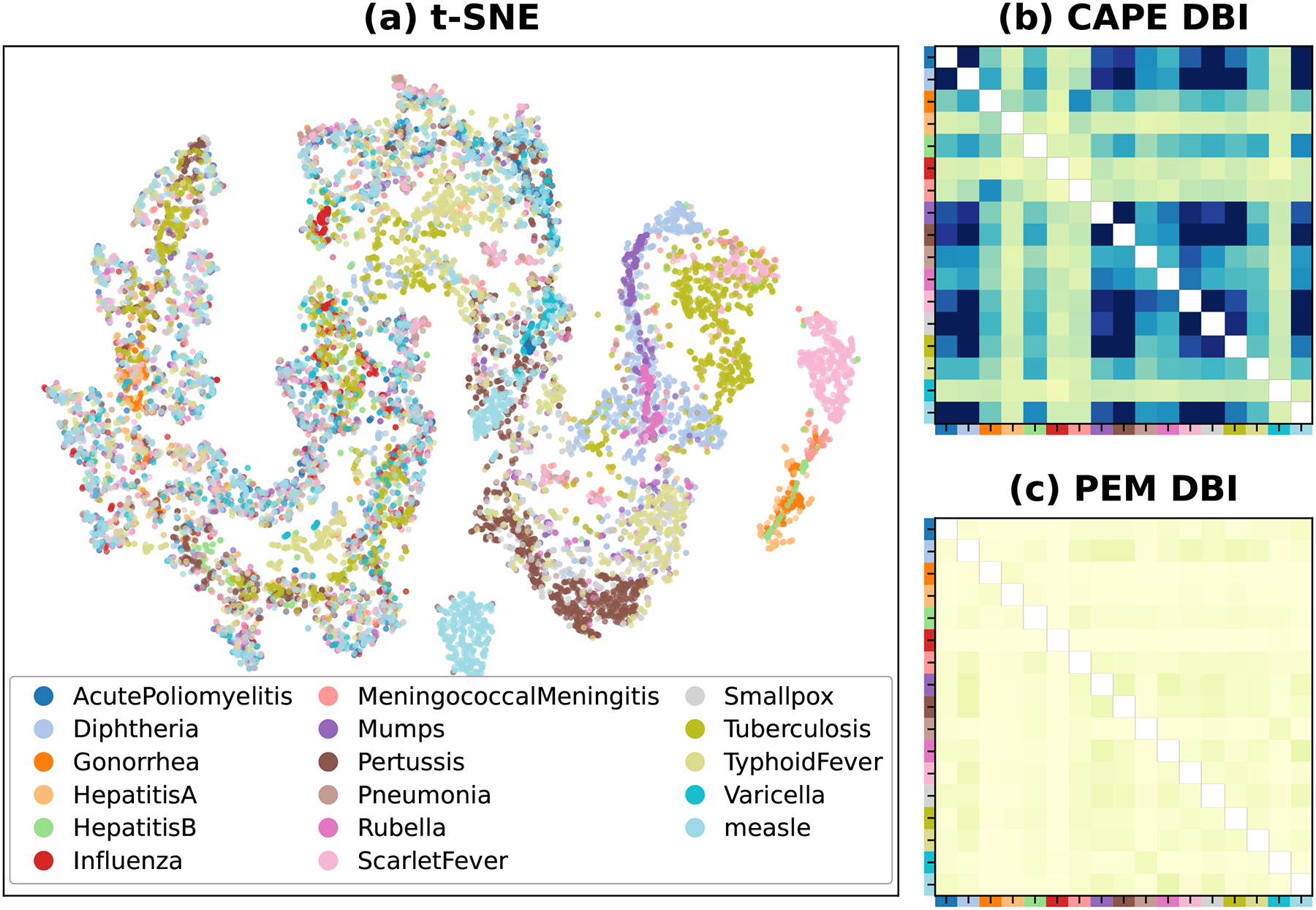
CAPE encodings of downstream diseases and pairwise DBI scores from CAPE and PEM (higher the darker).

Visual analysis via t-SNE (Figure 8a) reveals overlapping clusters, suggesting CAPE captures shared epidemic dynamics alongside disease-specific features. This balance is supported by pairwise Davies-Bouldin Index (DBI) analysis (Figures 8b–c), confirming CAPE learns both discriminative and shared patterns. Furthermore, disease classification results (Table 4) show that CAPE representations, unlike PEM, significantly improve non-linear classifier performance, demonstrating the model’s ability to encode complex non-linear correlations among diseases.

#### (d) Uncertainty Modeling via Pre-training.

**Figure 9:**
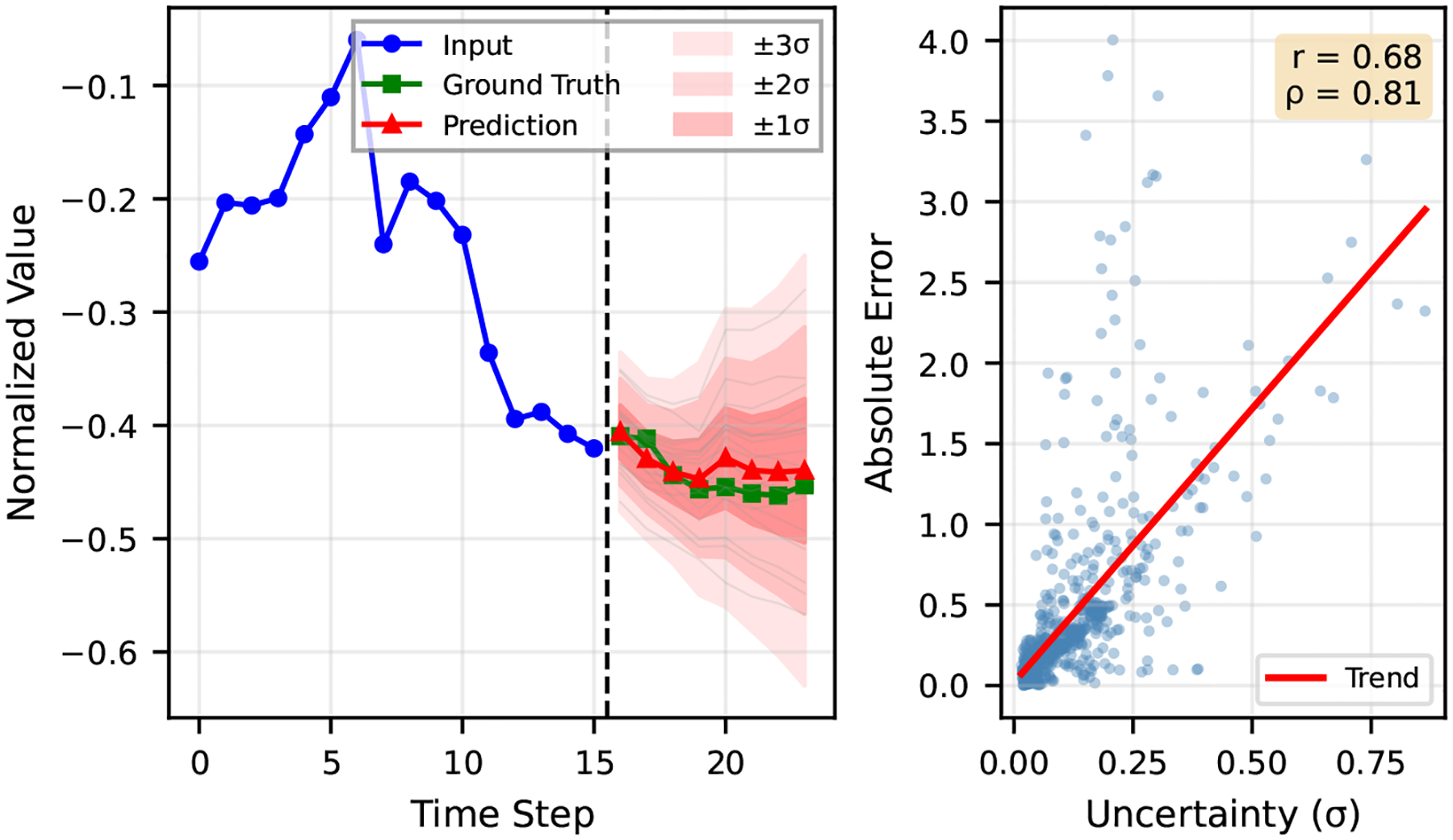
The strong positive correlations (Pearson r, Spearman ρ) indicate that higher predicted uncertainty corresponds to larger actual errors.

A key practical advantage of CAPE is that its compartmental masking doubles as a mechanism for uncertainty quantification at inference time, without any additional training. Because each compartment can be stochastically masked, a single forward pass corresponds to one plausible epidemic model structure, so repeating inference with different masks implicitly samples an ensemble of distinct compartmental models, mimicking how epidemiologists hedge across competing mechanistic assumptions [34]. We run K=50 masked passes (S and I are never masked), pool by their mean for the point forecast, and use their standard deviation as the uncertainty estimate. As shown in Figure 9, predicted uncertainty correlates strongly with actual error (Pearson r=0.68, Spearman ρ=0.81), confirming that CAPE reliably “knows when it does not know”—valuable for flagging low-confidence forecasts during emerging outbreaks.

## 5 Conclusion

We present CAPE, the first open-source pre-training framework for epidemic forecasting that learns flexible latent population states, termed compartmental prototypes, to address structural heterogeneity and hidden population states in epidemic pre-training. By designing a large-scale pre-training corpus with the proposed EpiRecipe simulation pipeline, CAPE captures generalizable dynamics across diseases. Across 17 diseases, CAPE outperforms strong full-shot baselines and general-purpose time series foundation models even in a zero-shot setting, while its compartmental masking yields calibrated uncertainty; our analysis further provides the first evidence of epidemic scaling laws, shows that pre-training *diversity* matters more than similarity to the target domain, and reveals epidemiologically meaningful representations learned without explicit supervision.

### Future Work.

Our results also point to several promising directions. First, EpiRecipe’s catalog of compartments and transition dynamics can be expanded to cover richer mechanisms, such as vector-borne transmission, multi-strain competition, and behavioral feedback, further broadening the diversity that drives transfer. Second, our current formulation is purely temporal; a natural extension is to spatiotemporal forecasting by replacing the temporal encoder with graph-based components that capture cross-region transmission. Finally, integrating textual and multimodal signals, such as surveillance reports, intervention policies, and non-pharmaceutical measures, would let the model reason over the contextual drivers of outbreaks, advancing toward trustworthy and actionable epidemic forecasting.

## Supplementary Material

Appendix

## Figures and Tables

**Figure 2:**
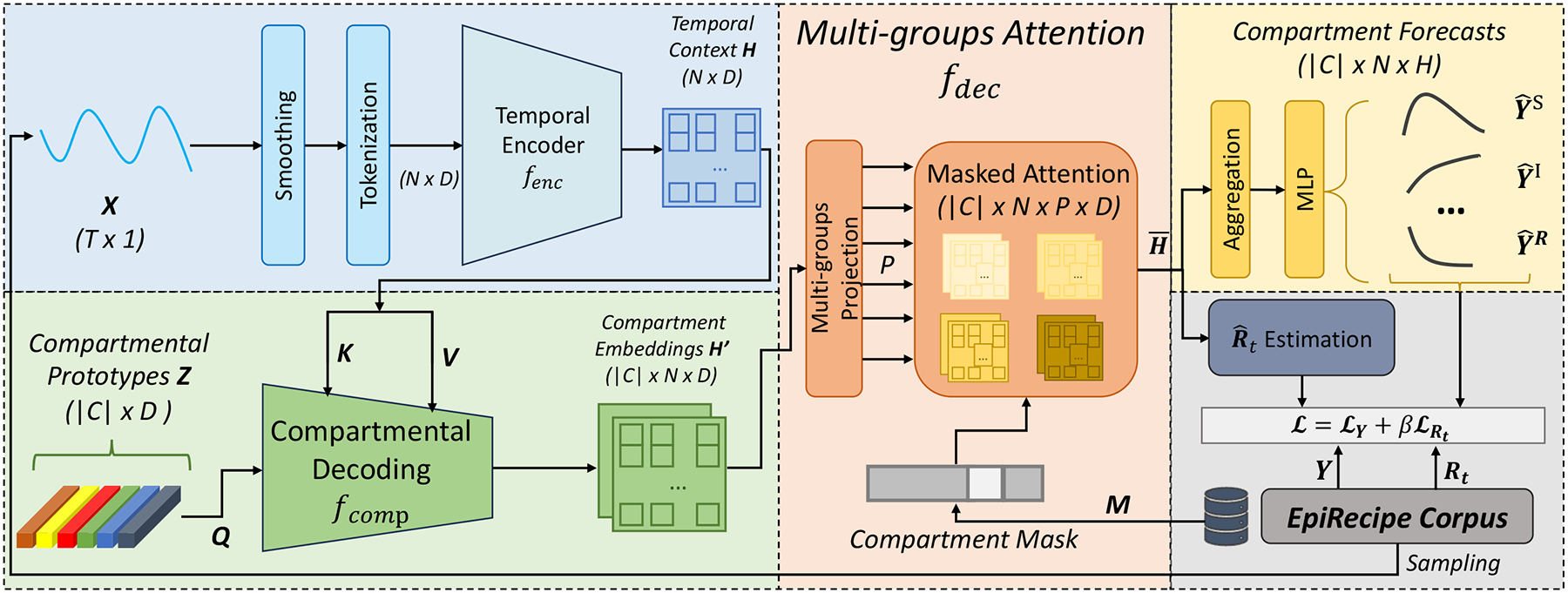
CAPE model structure and pre-training pipeline with EpiRecipe.

**Table 1:** Forecasting performance (MSE / MAE) across 17 diseases. Bold: best, underline: second best. First Count: number of diseases where CAPE beats the best baseline or ranks #1 among all models.

Disease	CAPE (Ours)				Baselines																	
	${\text{CAPE}}_{\text{zero}}$		CAPE		ARIMA		SIR		GRU		EINN		EpiDeep		DLinear		PatchTST		N-BEATS		PEM	
	MSE	MAE	MSE	MAE	MSE	MAE	MSE	MAE	MSE	MAE	MSE	MAE	MSE	MAE	MSE	MAE	MSE	MAE	MSE	MAE	MSE	MAE
Poliomyelitis	**0.235**_±0.002_	**0.226**_±0.001_	0.244_±0.000_	0.231_±0.001_	0.326	0.261	0.833	0.535	0.435	0.251	0.672	0.315	0.860	0.417	0.365	0.291	0.910	0.380	0.417	0.302	0.820	0.365
Diphtheria	0.168_±0.000_	0.196_±0.000_	0.163_±0.000_	**0.192**_±0.000_	**0.157**	0.211	0.437	0.478	1.008	0.551	1.859	0.735	1.322	0.636	1.200	0.546	2.591	0.790	2.720	0.874	1.115	0.549
Gonorrhea	0.060_±0.001_	0.138_±0.001_	0.059_±0.002_	0.139_±0.004_	**0.052**	**0.128**	0.652	0.758	0.116	0.241	1.760	0.788	0.877	0.784	0.157	0.304	1.333	0.752	1.077	0.659	0.333	0.390
Hepatitis A	0.207_±0.006_	0.318_±0.005_	**0.190**_±0.008_	**0.294**_±0.002_	0.228	0.324	0.740	0.702	0.253	0.350	0.489	0.535	0.679	0.693	0.267	0.382	0.683	0.667	0.514	0.560	0.863	0.741
Hepatitis B	**0.058**_±0.001_	0.146_±0.002_	0.058_±0.004_	0.145_±0.004_	0.061	**0.143**	0.254	0.409	0.083	0.208	0.087	0.213	0.269	0.442	0.094	0.238	0.156	0.286	0.238	0.303	0.211	0.359
Influenza	0.450_±0.008_	0.283_±0.018_	0.435_±0.001_	**0.191**_±0.001_	1.725	0.307	0.399	0.313	0.368	0.197	0.401	0.230	**0.261**	0.239	0.465	0.252	0.666	0.405	0.446	0.191	0.526	0.261
Meningitis	0.183_±0.024_	0.237_±0.009_	0.129_±0.000_	0.204_±0.001_	0.150	0.233	0.487	0.549	0.129	0.218	0.153	0.234	0.239	0.363	0.211	0.275	0.309	0.394	**0.103**	**0.203**	0.167	0.252
Mumps	0.019_±0.000_	0.062_±0.001_	**0.019**_±0.001_	**0.060**_±0.002_	0.025	0.068	0.249	0.475	0.046	0.154	0.024	0.102	0.156	0.373	0.058	0.208	0.054	0.181	0.046	0.160	0.075	0.217
Pertussis	**0.080**_±0.000_	**0.120**_±0.000_	0.085_±0.000_	0.123_±0.001_	0.109	0.142	0.226	0.376	1.258	0.590	2.030	0.760	1.514	0.681	0.756	0.434	1.775	0.708	2.149	0.753	1.716	0.701
Pneumonia	**0.043**_±0.000_	**0.118**_±0.000_	0.043_±0.000_	0.120_±0.000_	0.057	0.141	0.201	0.392	0.097	0.210	0.081	0.167	0.128	0.282	0.130	0.294	0.130	0.230	0.091	0.226	0.115	0.202
Rubella	0.025_±0.001_	0.080_±0.001_	**0.023**_±0.000_	**0.074**_±0.001_	0.029	0.083	0.239	0.469	0.278	0.331	0.090	0.240	0.600	0.713	0.131	0.320	2.224	1.161	1.679	0.776	1.325	0.875
Scarlet Fever	0.092_±0.000_	0.188_±0.000_	**0.088**_±0.000_	0.184_±0.000_	0.092	**0.178**	0.273	0.425	0.263	0.330	1.154	0.659	1.707	0.836	0.339	0.378	0.750	0.530	2.159	0.948	1.325	0.735
Smallpox	0.146_±0.000_	0.194_±0.000_	**0.142**_±0.001_	**0.192**_±0.000_	0.170	0.213	0.382	0.424	0.605	0.396	1.451	0.613	0.915	0.488	0.694	0.405	0.976	0.522	0.733	0.418	0.780	0.439
Tuberculosis	0.207_±0.003_	0.294_±0.002_	0.192_±0.002_	0.307_±0.003_	**0.173**	**0.268**	0.328	0.420	0.858	0.622	1.816	0.876	1.264	0.753	0.329	0.395	1.328	0.735	1.855	0.865	1.549	0.830
Typhoid Fever	0.485_±0.000_	0.320_±0.000_	0.473_±0.001_	0.318_±0.001_	**0.330**	**0.308**	0.705	0.575	0.635	0.411	1.491	0.622	1.268	0.578	0.896	0.475	0.810	0.479	10.26	1.299	1.716	0.709
Varicella	0.076_±0.000_	**0.144**_±0.000_	**0.076**_±0.000_	0.145_±0.001_	0.086	0.154	0.206	0.403	0.322	0.386	0.476	0.423	0.567	0.571	0.491	0.512	0.591	0.535	0.642	0.582	0.611	0.603
Measles	0.207_±0.001_	**0.206**_±0.000_	**0.206**_±0.001_	0.209_±0.001_	0.230	0.208	0.652	0.515	1.127	0.470	1.530	0.581	1.358	0.547	1.072	0.548	1.277	0.524	1.635	0.612	1.224	0.487
**Average**	0.161	0.192	**0.154**	**0.184**	0.235	0.198	0.427	0.483	0.464	0.348	0.915	0.476	0.823	0.553	0.450	0.368	0.974	0.546	1.575	0.572	0.851	0.513
**1st Count**	11	10	11	10	4	5	0	0	0	0	0	0	1	0	0	0	0	0	1	1	0	0

**Table 2:** Ablation Study Results (MSE ± Std). We conduct evaluations on the full data without train/val/test splits. Subscripts show relative improvement. Note that both pre-training methods are compared against the w/o pre-training baseline.

Method	Influenza	Diphtheria	Gonorrhea	Poliomyelitis	Tuberculosis	Rubella	Avg
w/o Pre-training	0.805_±0.004_	2.048_±0.003_	4.018_±0.002_	1.747_±0.003_	2.171_±0.003_	1.135_±0.006_	1.987
+ Pre-training (Real-world)	0.835_±0.032↑3.8%_	1.674_±0.032↓18.3%_	0.491_±0.032↓87.8%_	1.685_±0.032↓3.6%_	1.619_±0.032↓25.4%_	0.116_±0.032↓89.8%_	1.070_↓46.2%_
Pre-training (Naive Synth)	0.803_±0.039↓0.3%_	1.481_±0.043↓27.7%_	0.768_±0.039↓80.9%_	1.572_±0.042↓10.0%_	1.379_±0.040↓36.5%_	0.277_±0.041↓75.6%_	1.047_↓47.3%_
+ Comp. Decoding	0.678_±0.100↓15.6%_	0.549_±0.066↓62.9%_	0.059_±0.020↓92.3%_	0.448_±0.097↓71.5%_	0.603_±0.086↓56.3%_	0.064_±0.048↓76.8%_	0.400_↓61.8%_
+ Multi-groups Attn.	**0.652**_±0.112↓3.7%_	**0.547**_±0.093↓0.5%_	**0.046**_±0.025↓22.4%_	**0.463**_±0.090↑3.2%_	**0.450**_±0.092↓25.3%_	**0.068**_±0.040↑5.9%_	**0.371**_↓7.3%_

**Table 3:** Detailed statistics of downstream datasets from Project Tycho. “Len.” is the number of weeks after aggregating across states.

Disease	St.	Len.	Trans.	$R_{0}$	Disease	St.	Len.	Trans.	$R_{0}$
Measles	50	6,373	Resp.	12–18	Influenza	42	1,673	Resp.	1.2–1.6
Diphtheria	46	6,191	Resp.	1.7–4.3	Varicella	30	1,571	Resp.	10–12
Typhoid Fever	44	5,816	Fecal	2.8–7.0	Hepatitis B	31	1,449	Blood	1.0–3.3
Pertussis	46	5,719	Resp.	12–17	Rubella	7	1,427	Resp.	3.4–7.0
Scarlet Fever	48	4,957	Resp.	0.6–2.0	Mening.	37	1,330	Resp.	0.6–1.6
Tuberculosis	39	4,881	Resp.	0.24–4.3	Gonorrhea	39	1,126	Sexual	1.0
Smallpox	44	3,122	Resp.	3.5–6.0	Hepatitis A	38	1,096	Fecal	1.1–3.5
Ac. Poliomyelitis	47	2,534	Fecal	5–7	Mumps	41	2,419	Resp.	4–7
Pneumonia	41	2,060	Resp.	1.4					

**Table 4:** Disease Classification (17 disease labels) with linear and non-linear classifiers. (Acc. / F1)

Model	Random	Logistic Reg.	Ridge	KNN	Random Forest
CAPE	5.9 / 6	22.1 / 9.8	23.3 / 11.8	55.6 / 50.8	**62.7 / 57.3**
PEM	5.9 / 6	56.6 / 51.8	48.6 / 39.1	64.8/61.3	**72.9 / 68.6**

## CCS Concepts

• **Applied computing** → *Health informatics*.

## Footnotes

1
https://github.com/nuuuh/CAPE
