# Supplementary material for: Pre-training Epidemic Time Series Forecasters with Compartmental Prototypes
Source: KDD. Author manuscript; Available in PMC 2026 Aug 13. (PMC13464487; doi:10.1145/3770855.3818928)
Supplement: Supplementary file 1 [file NIHMS2195068-supplement-Appendix.pdf]

A Appendix

A.1 Limitations and Ethical Considerations

We use publicly available data without ethical concerns. While CAPE shows strong performance, it has several limitations. First, the compartment model combinations defined in EpiRecipe can be further expanded. Second, its effectiveness may be constrained by the scale and diversity of available data.

A.2 EpiRecipe

EpiRecipe is a constraint-based pipeline for generating diverse, epidemiologically valid compartmental models via automatic dependency resolution, rule-driven transition construction, hierarchical parameter sampling, and validation. It supports 12 compartment types, 28 transition rules across 14 categories, and population stratification with configurable mixing patterns.

A.2.1 Compartment Library. EpiRecipe supports 12 compartment types (Table 5), where each $X(t)$ is the number of individuals in state X at time t and N is the total population. Constraint-based selection first includes the mandatory $\{\mathbf{S}, \mathbf{I}\} \subseteq C^*$, then iteratively adds optional compartments while resolving dependencies, ensuring $\forall c \in C^* : f_{\text{req}}(c) \subseteq C^*$.

Table 5: Compartment types and structural constraints. Required compartments (S, I) are always included; optional compartments are sampled with automatic dependency resolution. "Cons." indicates population-conserved compartments.

Sym.	Type	Req.	Cons.	Sym.	Type	Req.	Cons.
S	Susceptible	✓	✓	D	Deceased	–	–
I	Infectious	✓	✓	V	Vaccinated	–	✓
E	Exposed	–	✓	Q	Quarantined	–	✓
R	Recovered	–	✓	W	Reservoir	–	–
A	Asymptomatic	–	✓	C	Chronic	–	✓
H	Hospitalized	–	✓	P	Prophylaxis	–	✓

Population Conservation: The "living" compartments from $\mathcal{L}$ satisfy $\sum_{X\in\mathcal{L}} X(t) = N - D(t)$ ($D(t)$ = cumulative deaths); the reservoir W (environmental pathogen load) is excluded.

A.2.2 Transition Rules. EpiRecipe implements 28 transition rules across 14 dynamics categories (Table 6), each specifying a flow rate between compartments.

A.2.3 Hierarchical Parameter Sampling. Parameters are sampled in three tiers for epidemiological consistency. **Tier 1** draws core quantities from informed priors: $R_0 \sim \text{LogNormal}(2.5, 0.8)$, infectious period $T_{\text{inf}} \sim \text{Gamma}(3, 3)$, and (if E present) latent period $T_{\text{lat}} \sim \text{Gamma}(2, 2.5)$, each clipped to a plausible range. **Tier 2** derives rates deterministically ($\gamma = 1/T_{\text{inf}}$, $\sigma = 1/T_{\text{lat}}$, $\beta = R_0\gamma$). **Tier 3** samples the 24 transition-specific rates (e.g., hospitalization, mortality, waning) uniformly within plausible ranges; full ranges are in the released code. Seasonal forcing is applied to 50% of models via $\beta(t) = \beta_0(1+\varepsilon\cos(2\pi t/T+\phi))$. Every model is then validated (population conservation, no dead-ends, a valid $S \to \{I, A, C\}$ pathway, and numerical stability), and failures are regenerated.

A.3 Population Stratification

Each group is defined by a population fraction and contact and susceptibility multipliers, using *Age* (children/adults/elderly) or *Risk* (low/high) presets. Inter-group contacts follow a row-stochastic mixing matrix $\mathbf{M}$ with homogeneous, assortative, or hierarchical structure (exact multipliers and matrices in the released code). **Simulation:** validated models are integrated by RK4 ($\Delta t = 1.0$, 250 steps, $N \in [10^3, 10^5]$) with Gaussian observation noise, yielding observables including incidence, prevalence, R_t, hospitalizations, and deaths.

Table 6: Complete transition dynamics catalog (28 rules across 14 categories). Representative rate-parameter ranges are listed in Appendix A.2.3.

Category	Rule	Flow Rate Equation
	mass_action	$\beta SI/N$
	with_asymptomatic	$\beta S(I+\epsilon A)/N$
	with_chronic	$\beta S(I+\delta_C C)/N$
Infection (7)	environmental	$\beta_W SW/(K+W)$
	combined_env	$\beta S(I+\rho W)/N$
	hospital_nosocomial	$\beta S(I+\theta H)/N$
	saturating	$\beta SI/(N(1+\alpha I))$
Latent prog. (2)	simple / branching	$\sigma E \to I$ / $\sigma E \to p_{\text{sym}} I + (1-p_{\text{sym}})A$
Recovery (2)	constant / resource-lim.	γX / $\gamma X/(1+\omega X)$, $X \in \{I, A, H\}$
Hospitaliz. (2)	constant / severity-dep.	ηI / $\eta I(1+\kappa I/N) \to H$
Death (2)	from I / from H	$\mu I \to D$ / $\mu_H H \to D$
Vaccination (2)	campaign / proportional	$\nu \to V$ / $p_{\text{vax}} S \to V$
Quarantine (2)	from I / from E	$\tau_Q I \to Q$ / $\tau_Q E \to Q$
Q release (2)	to R / to S	$\rho_Q Q \to R$ / $\rho_Q Q \to S$
Shedding (2)	from I / with asympt.	$\xi I \to W$ / $\xi(I+\delta_A A) \to W$
Env. decay (1)	decay	$-\zeta W$
Chronic (2)	progress / clear	$\chi I \to C$ / $\gamma_C C \to R$
Waning (2)	immunity / vaccine	$\omega R \to S$ / $\omega_V V \to S$

A.4 Theoretical Analysis

A.4.1 Estimation of R_t. We build the $\mathbf{F}$ (transmission) and $\mathbf{V}$ (removal) matrices from the I-to-I attention submatrix $A^{(I)}$ following the next-generation matrix framework [9], with $R_t = \rho(\mathbf{FV}^{-1})$. Off-diagonal entries $A^{(I)}_{ij}$ parallel cross-group transmission β_{ij}, so we form $\mathbf{F}$ by zeroing the diagonal; diagonal entries $A^{(I)}_{ii}$ (self-attention) reflect state persistence, analogous to the infectious period $1/\gamma$, so we set $\gamma_i = 1/(A^{(I)}_{ii} + \epsilon)$. This mirrors $R = \beta/\gamma$, and spectral bounds on $\mathbf{FV}^{-1}$ yield time-varying R_t estimates without explicit compartmental supervision.

A.4.2 Proof of spectral bounds in Algo. 1. **Setup and Preliminaries.** We write $\|M\|_2 = \sup_{x\neq 0} \|Mx\|_2/\|x\|_2 = \sigma_{\max}(M)$ for the operator 2-norm, with singular values the nonnegative square roots of the eigenvalues of M^*M, so that $\sigma_{\min}(M)\|x\|_2 \le \|Mx\|_2 \le \sigma_{\max}(M)\|x\|_2$ for any x. For square M, since $\prod_i \sigma_i(M) = |\det M| = \prod_i |\lambda_i(M)|$, comparing geometric means to extrema gives $\sigma_{\min}(M) \le (\prod_i |\lambda_i(M)|)^{1/n} \le \rho(M)$ (P1), and hence

$$\sigma_{\min}(M) \;\le\; \rho(M) \;\le\; \sigma_{\max}(M) = \|M\|_2. \qquad \text{(P2)}$$

Theorem A.1 (Bounds for R_t). *Let $F, V \in \mathbb{C}^{n\times n}$ with V invertible and $R_t \equiv \rho(FV^{-1})$, where $\rho(\cdot)$ is the spectral radius. Then $\frac{\sigma_{\min}(F)}{\sigma_{\max}(V)} \le \rho(FV^{-1}) \le \frac{\sigma_{\max}(F)}{\sigma_{\min}(V)}$.*

Proof. *Lower bound.* For any compatible A, B and unit vector x, $\|ABx\|_2 \ge \sigma_{\min}(A)\|Bx\|_2 \ge \sigma_{\min}(A)\sigma_{\min}(B)\|x\|_2$, hence $\sigma_{\min}(AB) \ge \sigma_{\min}(A)\sigma_{\min}(B)$. Applying this with $A = F$, $B = V^{-1}$ and using $\sigma_{\min}(V^{-1}) = 1/\sigma_{\max}(V)$ gives $\sigma_{\min}(FV^{-1}) \ge \sigma_{\min}(F)/\sigma_{\max}(V)$. Combined with (P1), $\sigma_{\min}(FV^{-1}) \le \rho(FV^{-1})$, this yields the lower bound $\sigma_{\min}(F)/\sigma_{\max}(V) \le \rho(FV^{-1})$.

Upper bound. For any square M, $\rho(M) \leq \|M\|_2$ by (P2), so $\rho(FV^{-1}) \leq \|F\|_2 \|V^{-1}\|_2 = \sigma_{\max}(F)/\sigma_{\min}(V)$. Together the two bounds give $\frac{\sigma_{\min}(F)}{\sigma_{\max}(V)} \leq \rho(FV^{-1}) \leq \frac{\sigma_{\max}(F)}{\sigma_{\min}(V)}$. □

A.5 Pre-train and Evaluation Datasets

A.5.1 Pre-training. In the first stage, CAPE is pretrained on synthetic epidemic time series from the EpiRecipe pipeline (Appendix A.2), which yields effectively unlimited data with known ground-truth compartmental structures. Each model randomly selects 3–7 compartments (S and I mandatory), and its compartments, transitions, parameters (e.g., R_0, T_{inf}), seasonal forcing, and group stratification are sampled as detailed in Appendix A.2. Each synthetic epidemic uses a population size in $[10^4, 10^6]$ and a length of 52–260 weeks at weekly resolution. To diversify periodic patterns, 20% of samples are instead generated from Gaussian Process priors (KernelSynth [3]) combining periodic, RBF, linear, and composite kernels. Samples are produced on-the-fly via streaming generation, 64 per batch over roughly 80,000 batches. In the second stage, we continue pre-training on 8 real-world diseases: Pertussis, Varicella, Tuberculosis, Measles, Typhoid Fever, Mumps, Diphtheria, and Scarlet Fever.

A.5.2 Downstream Evaluation Datasets. We evaluate CAPE on 17 real-world disease datasets from Project Tycho [40], a database of historical disease incidence in the United States. For each disease, weekly case counts are aggregated across all reporting states into a single national-level series (avoiding spatial modeling) and then z-score normalized prior to tokenization.

Online Evaluation Protocol. We adopt an online (rolling) setting [35]: the first 30% of the tokenized series is the initial training window, and the remaining 70% is split into $K = 2$ non-overlapping folds. Each fold uses an expanding training window (last 20% held for validation) to predict 1 output token (4 weeks) from 8 input tokens (32 weeks), respecting temporal order while evaluating across multiple epidemic phases. Per-dataset statistics (Table 3) show the diseases are highly heterogeneous, spanning 21–122 years (1888–2017), 1,096–6,373 weeks, respiratory/sexual/fecal-oral/bloodborne transmission, and R_0 from ≈ 1 (gonorrhea) to 12–18 (measles, pertussis).

A.6 Implementation Details

Model Details. We focus on temporal (rather than spatiotemporal) pre-training, which is largely unexplored [15] and data-efficient when reliable spatial graphs are unavailable; the framework extends to the spatiotemporal setting via graph-based encoders (future work). CAPE stacks 4 encoder layers (hidden size 512, 4 heads); each compartment is a learnable size-512 embedding mapped to 6 groups, and the patch encoder is a decoder-only transformer with causal masking, GELU activations, and sinusoidal positional encoding.

Pre-training. Stage 1 trains on EpiRecipe synthetic data (5,000 validation and 5,000 test samples spanning SIR/SEIR/SEIR-V dynamics) with token size 4, AdamW (lr 1×10^{-4}, weight decay 1×10^{-3}), batch size 64, a CosineAnnealingWarmRestarts scheduler (T_0=10, T_{mult}=2), loss weights α=1 for the I compartment and 0.5 otherwise with β=0.5, for 150 epochs. Stage 2 continues from the stage-1 checkpoint on the first 20% of each disease's series in chronological order (10% held for validation, ensuring temporal separation from evaluation data), with AdamW (lr 1×10^{-4}, weight decay 1×10^{-4}), batch size 32, Huber loss (δ=1.0), and 15 epochs, supervising only the I compartment and selecting the best checkpoint by validation loss. Both stages use 8 NVIDIA A100-80GB GPUs.

Table 7: The 14 ensemble pooling strategies, ordered conservative to aggressive. Here $\alpha = \max(0.3, 1 - \sigma/\bar{y})$ (variance_adaptive), $w_m \propto \hat{y}^{(m)}$ (optimistic_weighted), and $r^{(m)} = \hat{y}^{(m)} - y_{\text{last}}$ with $\alpha \in \{0.2, 0.3, 0.4\}$ (residual_correction).

Strategy	Estimate
percentile_75	P_{75}
median_max_blend	$0.5\,\text{med} + 0.5\,\text{max}$
upper_half_mean	$\bar{y}_{\text{upper}}$
biased_mean_50	$0.5\,\bar{y} + 0.5\,\text{max}$
optimistic_weighted	$\sum_m w_m \hat{y}^{(m)}$
robust_optimal	$\max(P_{75}, \bar{y}_{\text{upper}}, \hat{y}_{\text{optim}})$
adaptive_percentile	$P_q,\ q$=0.7+0.2 $\min(1, \frac{\sigma}{\bar{y}})$
trimmed_upper	mean of top 70%
quantile_blend	$0.5P_{60} + 0.5P_{80}$
shrinkage_upper_50	$0.5\,\bar{y}_{\text{upper}} + 0.5\,\text{med}$
variance_adaptive	$\alpha\,\text{med} + (1-\alpha)\,\text{max}$
max_plus_std	$\text{max} + \sigma$
beyond_max_20	$\text{max} + 0.2(\text{max} - \text{min})$
residual_correction	$y_{\text{last}} + \alpha e^{-\text{CV}(\mathbf{r})}\,\text{med}(r^{(m)})$

PEM Pre-training. For the PEM baseline [15], we pre-train a PatchTST-based model (d_{model}=256, 6 layers, 8 heads, patch length 4, dropout 0.1) by self-supervised masked prediction on all 17 diseases (first 30% of each series), combining RANDMASK (40%), LASTMASK (30%), and PEAKMASK (30%) at masking ratio γ=0.3, with AdamW (lr 1×10^{-4}, weight decay 1×10^{-3}), batch size 64, for 25 epochs.

Ensemble Aggregation Strategies. Since underestimating an outbreak is riskier than moderate overestimation, our aggregation strategies carry an *upward bias* toward higher-predicting members. Given M predictions $\{\hat{y}^{(m)}\}$ (mean $\bar{y}$, std σ, percentile P_q, upper-half mean $\bar{y}_{\text{upper}}$), we use the 14 strategies in Table 7, selecting per fold the one with lowest validation MSE.
